# Cost-effectiveness of pembrolizumab plus chemoradiotherapy versus placebo plus chemoradiotherapy for high-risk locally advanced cervical cancer in Saudi Arabia: A model-based economic evaluation

**DOI:** 10.1097/MD.0000000000050990

**Published:** 2026-10-02

**Authors:** Ziyad S. Almalki, Ahmad A. Alamer, Shaheed K. Alhumaid, Abdulrahman A. Alsuhibani, Bandar S. Alghamdi, Meshal M. Alqahtani, Nawaf S. Alzahrani, Osama A. Alghamdi, Haitham H. Alanazi

**Affiliations:** a Department of Clinical Pharmacy, College of Pharmacy, Prince Sattam Bin Abdulaziz University, Al-Kharj, Riyadh, Saudi Arabia; b Pharmacy Affairs Administration, King Fahad Specialist Hospital, Dammam, Saudi Arabia; c Department of Pharmacy Practice, College of Pharmacy, Qassim University, Qassim Saudi Arabia.

**Keywords:** cervical cancer, chemoradiotherapy, KEYNOTE-A18, partitioned survival model, PD-1 inhibitor, pembrolizumab, Saudi Arabia, structured expert elicitation

## Abstract

**Background:**

Adding pembrolizumab to standard concurrent chemoradiotherapy (CCRT) has shown substantial survival benefits in newly diagnosed, high-risk locally advanced cervical cancer (LACC), but its economic value in Saudi Arabia has not been established. This study assessed the cost-effectiveness of pembrolizumab plus CCRT versus placebo plus CCRT as first-line treatment for high-risk LACC from the perspective of the Saudi Arabian healthcare payer.

**Methods:**

A partitioned survival analysis model was developed to project lifetime costs and quality-adjusted life-years (QALYs) for a hypothetical cohort of women with high-risk LACC in the Saudi public healthcare sector, using resource use patterns consistent with major tertiary care and specialized cancer centers. Clinical efficacy data were obtained from the KEYNOTE-A18 trial. Costs were sourced from official Saudi price lists, local hospital data, and a formal Structured Expert Elicitation process, and were reported in 2025 United States dollars. Health state utilities were derived from the literature. The primary outcome was the incremental cost-effectiveness ratio. Future costs and QALYs were discounted at an annual rate of 3%. Comprehensive deterministic and probabilistic sensitivity analyses were conducted to assess uncertainty.

**Results:**

Adding pembrolizumab to CCRT resulted in an incremental gain of 3.51 life-years and 2.93 QALYs at an additional cost of $242,043 compared with placebo plus CCRT. At its current list price, the regimen yielded an incremental cost-effectiveness ratio of $82,626 per QALY. At a willingness-to-pay threshold of $90,000 per QALY, the probability of the pembrolizumab regimen being cost-effective was 67%.

**Conclusion:**

Pembrolizumab in combination with CCRT provides substantial clinical benefits in patients with high-risk LACC in Saudi Arabia. At the current price, this regimen is likely to be considered cost-effective. The analysis provides a strong, evidence-based rationale for significant price negotiations to ensure that the regimen delivers value to the Saudi healthcare system.

## 1. Introduction

Cervical cancer is a growing public health concern in Saudi Arabia.^[1]^ Current estimates indicate that 358 new cases are diagnosed annually, leading to 179 deaths from the disease. The crude incidence rate is 2.44 per 100,000 women, with an age-standardized rate of 2.81, while the crude mortality rate is 1.22 per 100,000 women, with an age-standardized rate of 1.60.^[2]^ These figures establish cervical cancer as the 8^th^ most frequent cancer among women in Saudi Arabia, both overall and specifically within the 15 to 44 age group.^[3]^

A particularly troubling aspect of this issue is the high proportion of patients diagnosed at an advanced stage of the disease. Over 40% of cervical cancer cases in Saudi women are locally advanced cervical cancer (LACC).^[4]^ This disparity is largely attributed to the historical absence of a comprehensive national screening program and low uptake of existing opportunistic services. For instance, a 2023 study reported a screening prevalence of only 22.1% among Saudi females.^[4]^ Compounding this issue is the low adoption of the human papillomavirus vaccine, a key primary prevention strategy, with 1 study finding that only 8.25% of participants had been vaccinated.^[5]^ Consequently, a significant proportion of the cervical cancer patient population in Saudi Arabia presents with LACC, underscoring the critical need for effective treatments for this advanced stage of the disease.^[6]^

In line with international guidelines,^[7]^ the established standard of care for LACC in Saudi Arabia is concurrent chemoradiotherapy (CCRT). This regimen typically involves weekly cisplatin-based chemotherapy along with external beam radiotherapy, followed by intracavitary brachytherapy. Although CCRT has improved outcomes compared to radiotherapy alone, it is not always sufficient.^[8]^ Relapse rates for high-risk LACC can be as high as 50%,^[9]^ highlighting the significant unmet need for more effective therapeutic strategies to improve patient outcomes.

Addressing this therapeutic gap has emerged as a promising approach. Pembrolizumab, a humanized monoclonal antibody targeting the programmed death-1 immune checkpoint, has shown significant potential. By blocking the interaction between programmed death-1 and its ligands, Programmed Death-Ligand 1 and Programmed Death-Ligand 2, pembrolizumab enhances the body’s own T-cell mediated antitumor response.^[10]^ The efficacy of adding pembrolizumab to CCRT was evaluated in the KEYNOTE-A18/ European Network for Gynaecological Oncological Trials (ENGOT-cx11)/Gynecologic Oncology Group (GOG-3047) trial, a phase 3, randomized, double-blind, placebo-controlled study involving 1060 patients with high-risk LACC. This trial demonstrated a statistically significant and clinically meaningful improvement in progression-free survival (PFS). The results showed a substantial reduction in the risk of disease progression or death, a finding with a high degree of statistical confidence. After an extended follow-up period, a greater proportion of patients in the pembrolizumab arm were progression-free compared to those in the placebo arm. Emerging data also suggest an overall survival (OS) benefit, with higher survival rates observed in the pembrolizumab group versus the placebo group.^[11]^

Although the clinical benefits of pembrolizumab are promising, its substantial acquisition cost presents a significant challenge for health systems worldwide.^[12]^ Consequently, the landmark KEYNOTE-A18 trial spurred numerous economic evaluations; however, these have yielded a complex and often contradictory picture. For instance, 1 analysis suggested that the regimen is cost-effective in the United States and China but not in Italy.^[13]^ Other detailed studies have concluded the opposite, finding that the treatment price places it well beyond conventional cost-effectiveness thresholds in both the United States and China without significant discounts.^[14]^

This divergence in international findings illustrates that economic conclusions are not universally applicable. Such disparities are primarily driven by jurisdictional variations in local pharmaceutical pricing, differing costs of healthcare delivery (such as radiotherapy tariffs and inpatient care), variations in locally accepted willingness-to-pay (WTP) thresholds, and divergent methodological choices regarding long-term survival extrapolation. Consequently, a critical research gap exists, as no pharmacoeconomic analysis of this regimen for LACC has been conducted from a Saudi Arabian perspective. Such an analysis is a national priority, aligning directly with the principles of Saudi Vision 2030, which mandates a healthcare system focused on efficiency and value-based outcomes.^[15]^ Given the high proportion of patients in the Kingdom who present with advanced disease, clarifying the value of innovative first-line treatment has a particularly pronounced clinical and economic impact in this region. Therefore, this study aimed to assess the cost-effectiveness of adding pembrolizumab to standard CCRT as the first-line treatment for adult patients with newly diagnosed, high-risk LACC from the perspective of the Saudi Arabian healthcare payer.

## 2. Methods

### 2.1. Analytic framework and target population

This economic evaluation was designed and reported in accordance with the Consolidated Health Economic Evaluation Reporting Standards (CHEERS) 2022 statement to ensure methodological transparency and to facilitate critical appraisal.^[16]^ The analysis was conducted from the perspective of the Saudi Arabian healthcare payer and therefore included only direct medical costs.

The model simulated a hypothetical cohort of women aged ≥18 years with newly diagnosed high-risk LACC. To ensure the highest degree of clinical relevance and validity, the cohort’s baseline characteristics were directly informed by the intention-to-treat population of the pivotal, global, randomized, double-blind, phase 3 ENGOT-cx11/GOG-3047/KEYNOTE-A18 trial (NCT04221945). This trial established the efficacy and safety of adding pembrolizumab to the standard CCRT.^[11]^

In line with the trial’s eligibility criteria, high-risk LACC was defined as disease meeting the International Federation of Gynecology and Obstetrics 2014 criteria for Stage IB2-IIB with positive pelvic lymph nodes, or Stage III-IVA disease.^[17]^ A baseline median age of 50 years was used, reflecting the median age reported for patients in the KEYNOTE-A18.

### 2.2. Model structure and assumptions

To project lifetime costs and health outcomes, a hybrid economic model was developed using TreeAge Pro 2025 (TreeAge Software, LLC, Williamstown).^[18]^ The model adopted a hybrid structure: a short decision tree allocates the hypothetical cohort to 1 of the 2 competing treatment strategies at model entry, after which a partitioned survival analysis framework projects health outcomes and costs over the lifetime horizon. This framework was deliberately chosen because it is a common and widely accepted approach in oncology technology appraisals, directly utilizing the primary time-to-event endpoints of PFS and OS reported in pivotal trials such as KEYNOTE-A18. This direct alignment enhances the model’s transparency by eliminating the need to derive complex transition probabilities.

The model is structured around 3 mutually exclusive health states, with the proportion of the cohort in each state at any given time determined directly by survival curves. These states include pre-progression (patients who are alive and progression-free disease, defined by the PFS curve), post-progression (patients who are alive but have experienced progressive disease, defined by the area between the OS and PFS curves), and death (the final, absorbing state, defined as 1 minus the OS curve) (Fig. 1).

**Figure 1.**
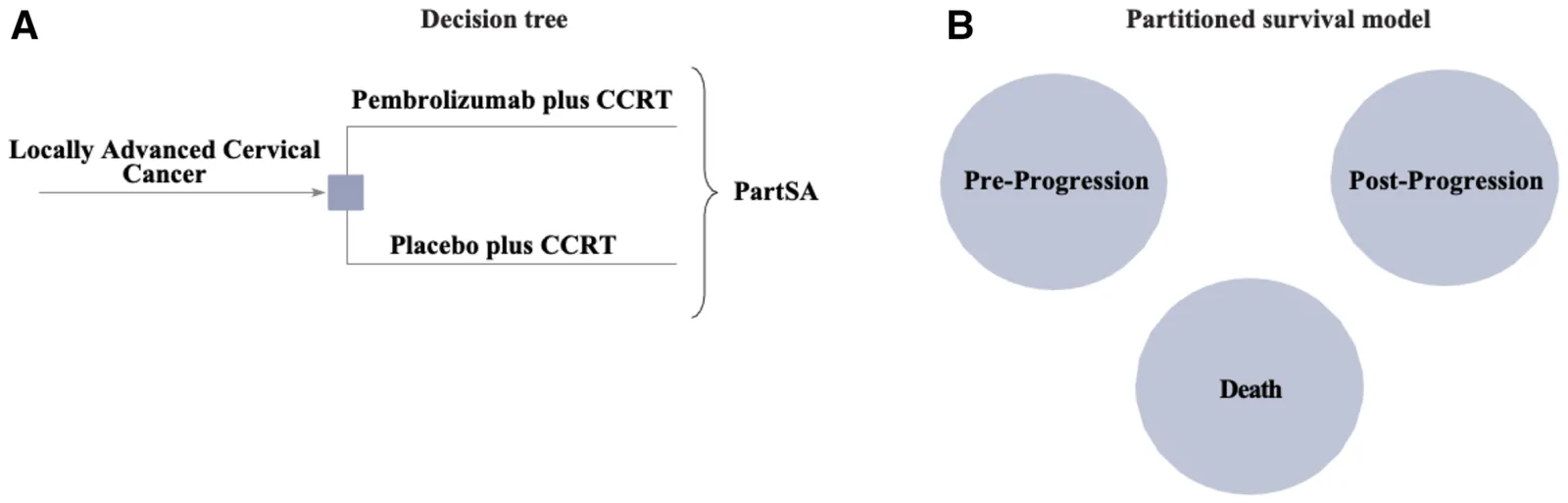
Structure of the hybrid economic model. (A) Decision tree structure depicting locally advanced cervical cancer pathways for pembrolizumab plus CCRT versus placebo plus CCRT. (B) Partitioned survival model framework comprising pre-progression, post-progression, and death states. CCRT = concurrent chemoradiotherapy.

A lifetime horizon of 50 years was adopted to capture the full lifetime costs and health benefits, consistent with international Health Technology Assessment (HTA) recommendations.^[19]^ The model operated on a 3-week cycle length to align with the trial’s treatment administration schedule, and future costs and quality-adjusted life-years (QALYs) were discounted at an annual rate of 3%. A half-cycle correction was applied to both costs and QALYs to provide a more accurate estimation of the lifetime outcomes.

### 2.3. Treatment strategies and subsequent therapies

The treatment pathways simulated in the model replicated the regimens used in the KEYNOTE-A18 trial.^[11]^ The intervention arm consisted of pembrolizumab (200 mg intravenous every 3 weeks for 5 cycles, then 400 milligram (mg) intravenous every 6 weeks for 15 cycles) administered with and after a standard CCRT regimen. The comparator arm received a placebo on the same schedule as the identical CCRT regimen. The CCRT backbone for both arms comprised weekly cisplatin ((40 milligrams per meter squared (mg/m^2^)) and a two-phase radiation protocol of External Beam Radiotherapy, predominantly Intensity-Modulated Radiotherapy (45 gray (Gy) in 25 fractions (1.8 Gy/fraction) to the pelvis) followed by high-dose rate brachytherapy (28 Gy in 4 fractions (7 Gy/fraction) to High-Risk Clinical Target Volume).

A critical component of the model is the asymmetric pathway for subsequent therapies, which reflects the current evidence-based clinical practice. Upon progression, patients in the placebo arm, being immunotherapy-naïve, were assumed to receive the established first-line standard for metastatic disease: the KEYNOTE-826 regimen (pembrolizumab plus platinum-based chemotherapy, with or without bevacizumab).^[20]^ Conversely, patients who progressed after receiving first-line pembrolizumab were assumed to receive a platinum-based chemotherapy doublet with or without bevacizumab.

### 2.4. Clinical efficacy and survival analysis

The clinical efficacy inputs for PFS and OS were derived from the final analysis of the KEYNOTE-A18 trial. In the absence of individual patient data, a rigorous multi-step process was employed to reconstruct the time-to-event data. First, the published Kaplan–Meier curves were digitized using the validated tool WebPlotDigitizer (version 4.7).^[21]^ Second, the digitized coordinates and published numbers at risk were used as inputs for the iterative algorithm developed by Hoyle and Henley to generate a pseudo-individual patient data dataset, which provides a more accurate representation of the underlying survival data than traditional curve-fitting.^[22]^ Third, to project long-term outcomes, we fitted a comprehensive suite of parametric survival models (including Exponential, Weibull, Gompertz, Log-logistic, Log-normal, and Generalized Gamma) to the reconstructed trial data. The final models for extrapolation were selected by balancing 2 key factors: their statistical goodness-of-fit, as measured by the Akaike Information Criterion (AIC) and Bayesian Information Criterion (BIC) (Table 1), and clinical plausibility, confirmed through visual inspection (Fig. 2).

**Table 1:** Akaike information criterion and Bayesian information criterion statistics for alternate parametric survival distributions.

Population group	Distribution	Pembrolizumab plus CCRT (AIC)	Pembrolizumab plus CCRT (BIC)	Placebo plus CCRT (AIC)	Placebo plus CCRT (BIC)
**OS**	Exponential	783.5256	786.4159	893.4643	896.4201
	Weibull	767.5074	773.2881	880.4385	886.3501
	Gompertz	765.7435	771.5242	881.6312	887.5429
	logistic	792.2025	797.9832	915.279	921.1907
	Log-normal	792.3802	798.1609	891.3517	897.2633
	Log-logistic	777.2913	783.072	886.8258	892.7375
	Generalized Gamma	767.2598	775.9308	882.2138	891.0813
**PFS**	Exponential	1054.393	1057.558	1084.019	1087.178
	Weibull	1034.645	1040.975	1068.052	1074.37
	Gompertz	1032.814	1039.144	1071.465	1077.783
	logistic	1072.683	1079.013	1113.161	1119.479
	Log-normal	1054.435	1060.764	1074.795	1081.113
	Log-logistic	1046.106	1052.435	1072.603	1078.921
	Generalized Gamma	1030.961	1040.455	1069.979	1079.457

**Figure 2.**
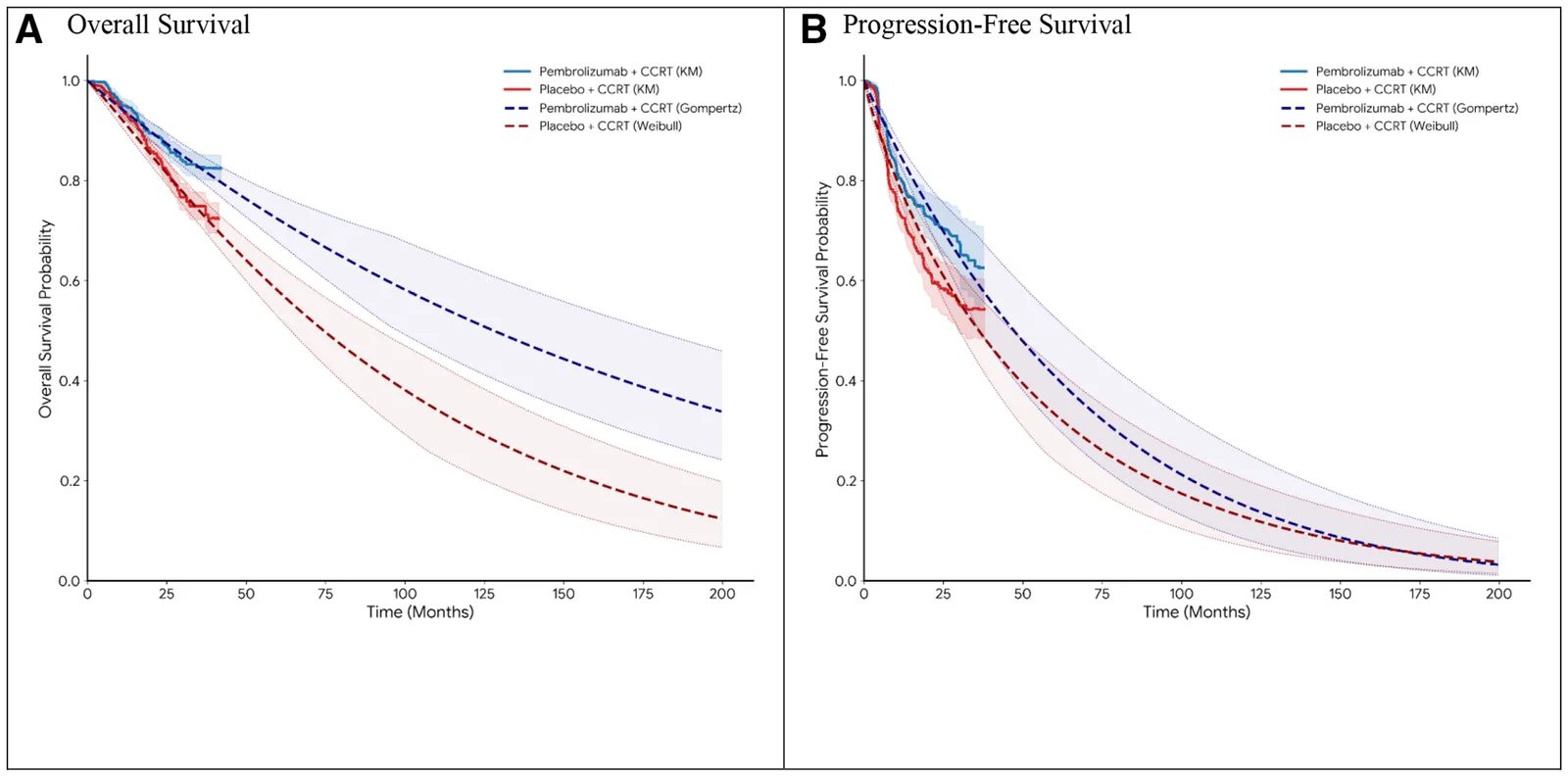
Kaplan-Meier curves and long-term extrapolations of survival outcomes from KEYNOTE-A18. (A) Overall survival curves and parametric extrapolations. (B) Progression-free survival curves and parametric extrapolations. CCRT = concurrent chemoradiotherapy, KM = Kaplan–Meier.

For the OS data, the Gompertz distribution provided the best statistical fit for the pembrolizumab arm (AIC: 765.7435; BIC: 771.5242), which was superior to the Generalized Gamma (AIC: 767.2598) and Weibull (AIC: 767.5074). This statistical superiority indicates that the Gompertz distribution most accurately captures the hazard rate observed in the trial data. For the placebo arm, the Weibull distribution offered the best fit (AIC: 880.4385; BIC: 886.3501), outperforming the Log-logistic (AIC: 886.8258) and Gompertz (AIC: 881.6312) models.

For the PFS data, the Gompertz distribution was selected for the pembrolizumab arm (AIC: 1032.814) despite the Generalized Gamma distribution yielding a marginally lower score (AIC: 1030.961). Because an AIC difference of <2 indicates statistically indistinguishable models, the selection was ultimately driven by clinical plausibility. The highly flexible, 3-parameter Generalized Gamma model risks overfitting the relatively immature trial data. Meanwhile, the Weibull distribution was selected for the placebo arm (AIC: 1068.052) based on standard statistical minimization criteria.

Beyond immediate statistical fit, the Gompertz distribution was deemed uniquely suited for the pembrolizumab arm because it features a hazard function capable of modeling the potential for long-term survival plateaus a characteristic phenomenon where a subset of patients experiences durable disease control, as is often hypothesized with immune checkpoint inhibitors. Conversely, other models, such as the Log-normal or Log-logistic, risk overestimating long-term survival by projecting heavy tails that may not be clinically realistic for this population. This projection and model choice were rigorously validated by the clinical expert panel, who confirmed that the Gompertz projection aligned with their clinical expectations for long-term survivorship in responders.

Finally, to calculate all-cause mortality, disease-specific mortality from the fitted OS models was integrated with background mortality rates from the most recent official life tables for the general female population in Saudi Arabia, published by the general authority for statistics.^[23]^ Finally, the incidence rates of severe (grade 3 or higher) adverse events (AEs) occurring in 5% or more of patients were also sourced directly from the KEYNOTE-A18 trial publication. In addition, a quantitative internal validation was performed by comparing the survival rates predicted by our model at key time points against the observed data reported in the KEYNOTE-A18 publication. As detailed in Supplementary Table S1, Supplemental Digital Content 1, the model-predicted survival rates at 24 months (for PFS) and 36 months (for OS) are in close alignment with the trial’s reported outcomes, confirming the model’s numerical accuracy and predictive validity.

### 2.5. Costing and resource utilization

Consistent with the Saudi Arabian healthcare payer perspective, our analysis only included direct medical costs. All costs were sourced in May 2025 and reported in 2025 United States dollars (USD); therefore, no inflation adjustments were required. Costs were converted from Saudi Riyals at an official pegged exchange rate of 1 USD = 3.75 Saudi Riyals, as maintained by the Saudi Central Bank.^[24]^ Pharmaceutical acquisition costs were sourced from the official Saudi Food and Drug Authority price lists,^[25]^ while radiotherapy costs were based on official tariffs from King Abdulaziz Medical City (KAMC) in Riyadh. As a major national referral hospital, KAMC’s tariffs are considered a key benchmark for specialized care costs in the Saudi healthcare system.

To address the scarcity of published local cost data for AE management, routine follow-up (encompassing clinical consultations, routine laboratory tests, periodic diagnostic imaging, and drug administration), and terminal care, a formal structured expert elicitation (SEE) was conducted following the ISPOR Task Force Good Practice recommendations.^[26]^ SEE is a formal, scientific methodology for quantifying the subjective judgment of experts regarding an uncertain quantity in a transparent, replicable manner designed to minimize cognitive biases. This is particularly valuable in situations such as the present study, where empirical data are sparse. A multi-disciplinary panel of 17 Saudi-based clinical experts was convened (Table 2). Experts were selected based on a minimum of 5 years of post-specialization experience in managing cervical cancer patients within major Saudi tertiary care or cancer centers. The panel comprised 8 medical oncologists, 5 radiation oncologists, and 4 senior clinical pharmacists with expertise in oncology, ensuring all facets of resource use were captured. A two-round modified-Delphi process was used to achieve consensus. In the first round, experts individually provided 3-point estimates (minimum, most likely, maximum) for the resource utilization (e.g., physician time, hospital days, number of tests) associated with defined clinical scenarios. In the second round, anonymized, aggregated results from the first round were distributed to the panel, who were then asked to review their initial estimates and revise them if they felt it appropriate. This process was repeated until consensus (defined as a <15% change in the mean value between rounds) was achieved. These resource estimates were subsequently multiplied by national unit costs to derive the final cost inputs. To model the uncertainty inherent in these estimates, the 3-point estimates for resource use were used to parameterize beta-PERT distributions. The mean of each resulting cost distribution was used for the deterministic base-case analysis, while the full distributions were sampled in the probabilistic sensitivity analysis (PSA). Full summary statistics for the elicited parameters are provided in Supplementary Table S2, Supplemental Digital Content 2.

**Table 2:** Composition and rationale of the multi-disciplinary expert panel.

Specialty	Number of experts	Rationale for inclusion in the elicitation panel
**Medical Oncology**	8	To provide core estimates for physician time, inpatient stays, and outpatient visits related to the overall management of cancer patients and the most common adverse events.
**Radiation Oncology**	5	To ensure accurate estimation of resources specific to radiation treatment pathways and the management of toxicities unique to this modality.
**Senior Clinical Pharmacy (Oncology**)	4	To capture the “hidden” but substantial costs associated with pharmaceutical services, including specialized pharmacist time, drug wastage, and medication regimens.

### 2.6. Health-related quality of life and utilities

Health outcomes were measured in QALYs and calculated by multiplying the time spent in each health state by its corresponding health state utility value (HSUV). Owing to the lack of a country-specific EQ-5D value set for Saudi Arabia, HSUVs were sourced from a targeted literature review. The study by Park et al was selected as the most appropriate source for several reasons: it evaluated patients with cervical cancer, and its health states were defined by disease progression (pre-progression vs post-progression), which aligns directly with the model structure, and it utilized the well-established EuroQol 5-Dimension 3-Level instrument instrument to derive utility values.^[27]^ Although cultural differences are a limitation, these specific methodological similarities provide the most robust and clinically relevant values available. Based on this source, utility values of 0.87 and 0.78 were applied to the pre- and post-progression states, respectively. Temporary utility decrements for AEs were also applied to capture the transient negative impact of treatment, with values sourced from published studies.^[28,29]^

### 2.7. Economic analysis and uncertainty assessment

The primary outcome of the base-case analysis was the incremental cost-effectiveness ratio (ICER), expressed as cost in USD per QALY gained. In the absence of a formally established explicit WTP threshold in Saudi Arabia, the cost-effectiveness of the intervention was evaluated against an evaluative benchmark of $90,000 per QALY. This value, equivalent to approximately 2.8 times the national gross domestic product (GDP) per capita, was selected as a methodologically justified reference point, rather than as a definitive decision rule. It aligns with the upper end of the range (1–3 times GDP per capita) suggested by the World Health Organization,^[30]^ though it is recognized that these generalized thresholds are increasingly debated, especially for high-income countries.^[31]^ Nevertheless, it must be explicitly acknowledged that generalized GDP-based thresholds have become increasingly controversial in modern health economics. Contemporary HTA frameworks caution against the universal application of WHO-CHOICE multipliers, arguing they may not accurately reflect the true marginal opportunity costs of health systems and can lead to the displacement of highly efficient foundational care. While we acknowledge that broad GDP-based heuristics have these limitations, the selection of a multiplier near the 3x ceiling is methodologically justified by the specific clinical context: LACC is a severe, life-threatening condition with high unmet needs. This approach mirrors evolving HTA frameworks in the Gulf region, such as those in the United Arab Emirates, which explicitly incorporate multipliers of up to 3x GDP for rare or severe diseases to reflect societal preferences for prioritizing high-burden conditions. Ultimately, while the selected benchmark serves as a pragmatic evaluative tool, it underscores the uncertainty inherent in decision-making. To further explore the uncertainty around this value, additional sensitivity analyses were conducted.

To assess the impact of uncertainty on the results, a comprehensive set of analyses was conducted. A one-way deterministic sensitivity analysis identified the main drivers of the ICER, and the results are presented in a tornado diagram. This involved systematically varying each parameter across a plausible range using 95% confidence intervals, where available. In cases where empirical data for specific parameters were lacking, a conservative standardized range was applied: ±20% for probabilities and utilities and ±30% for costs. This approach is common and accepted in health economic modeling, ensuring that all influential parameters are examined. A PSA was conducted using a 5000-iteration Monte Carlo simulation, with results presented in the cost-effectiveness acceptability curves (CEACs) and scatter plots. The key parameters used to populate the model, including their base-case values, distributions for the sensitivity analysis, and sources, are summarized in Table 3.

**Table 3:** Key model parameters, values, and sources for the cost-effectiveness.

Parameter category	Parameter name	Base-case value, (e.g., mean)	Sensitivity analyses		Source
			Distribution type	SD/SE	
Population	Baseline Median Age (yr)	50	Fixed	–	Assumed
	Mean Body Surface Area (m^2^)	1.84	Fixed	–	^[28]^
Clinical efficacy	Gompertz survival model for OS (Pembrolizumab plus CCRT)	Scale Parameter (η): 0.00005 Shape Parameter (b): 0.0054	Fixed	–	Model fitting
	Gompertz survival model for PFS (Pembrolizumab plus CCRT)	Scale Parameter (η): 0.002 Shape Parameter (b): 0.014	Fixed	–	Model fitting
	Weibull survival model for OS (Placebo plus CCRT)	Scale Parameter (λ): 0.0210 Shape Parameter (k): 1.4973	Fixed	–	Model fitting
	Weibull survival model for PFS (Placebo plus CCRT)	Scale Parameter (λ): 0.0253 Shape Parameter (k): 2.2117	Fixed	–	Model fitting
Rate of severe events	Pembrolizumab plus CCRT				
	Anemia	0.3	Beta	0.031 (SE)	^[11]^
	Leukopenia	0.18	Beta	0.018 (SE)	^[11]^
	Neutropenia	0.12	Beta	0.013 (SE)	^[11]^
	Neutrophil count decreased	0.16	Beta	0.018 (SE)	^[11]^
	Placebo plus CCRT				
	Anemia	0.27	Beta	0.028 (SE)	^[11]^
	Leukopenia	0.16	Beta	0.018 (SE)	^[11]^
	Neutropenia	0.10	Beta	0.010 (SE)	^[11]^
	Neutrophil count decreased	0.14	Beta	0.013 (SE)	^[11]^
	Placebo plus CCRT	0.814	Beta	0.082 (SE)	^[11]^
Cost (US$. 2025)	Pembrolizumab	39.20 per mg	Gamma	5.88 (SE)	^[25]^
	Paclitaxel	1.25per mg	Gamma	0.19 (SE)	^[25]^
	Carboplatin	0.013 per mg	Gamma	0.002 (SE)	^[25]^
	Cisplatin	0.30 per mg	Gamma	0.045 (SE)	^[25]^
	Bevacizumab	1.86 per mg	Gamma	0.28 (SE)	^[25]^
	Intensity-Modulated Radiation Therapy (IMRT)	959.88 per session	Gamma	143.98 (SE)	KAMC
	Brachytherapy	719.91 per session	Gamma	110.19 (SE)	KAMC
	Management of severe events				
	Anemia	1371.15	Beta-PERT	221.00 (SD)	Structured Expert Elicitation
	Leukopenia	1108.02	Beta-PERT	114.00 (SD)	Structured Expert Elicitation
	Neutropenia	2076.54	Beta-PERT	169.00 (SD)	Structured Expert Elicitation
	Neutrophil count decreased	2076.54	Beta-PERT	147.00 (SD)	Structured Expert Elicitation
	Routine follow-up (per cycle)	685.35	Beta-PERT	121.49 (SD)	Structured Expert Elicitation
	Terminal Care	2256.45	Beta-PERT	278.22 (SD)	Structured Expert Elicitation
Utilities	Pre-progression (Progression-Free)	0.87	Beta	0.044 (SE)	^[27]^
	Post-Progression (Progressed Disease)	0.78	Beta	0.039 (SE)	^[27]^
	Disutility for severe events				
	Anemia	−0.051	Beta	0.005 (SE)	^[28]^
	Leukopenia	−0.045	Beta	0.005 (SE)	^[29]^
	Neutropenia	−0.059	Beta	0.006 (SE)	^[29]^
	Neutrophil count decreased	−0.059	Beta	0.006 (SE)	^[29]^

All costs are presented in 2025 United States Dollars ($) from a Saudi payer perspective.

AE = adverse event, KAMC = King Abdulaziz Medical City, OS = overall survival, PFS = progression-free survival, SD = standard deviation, SE = standard error.

To evaluate the uncertainty and broader economic impact of the intervention, additional scenario analyses were conducted. First, a societal perspective was explored to capture indirect costs associated with productivity loss. These were estimated using the human capital approach, utilizing age-specific female labor force participation rates and average wage data from general authority for statistics. Second, to assess the model’s reliance on subjective data, a robustness check was performed by excluding all costs derived from the SEE process. Finally, a threshold price analysis was calculated to determine the percentage reduction in the acquisition cost of pembrolizumab required to meet stricter alternative efficiency benchmarks, specifically $50,000 per QALY and $20,000 per QALY.

### 2.8. Ethical approval

This study is a model-based economic evaluation that relies on publicly available data from previously published clinical trials (KEYNOTE-A18) and other literature sources; however, the SEE process involved the participation of human healthcare professionals. The study was submitted to the Institutional Review Board of the Riyadh Second Health Cluster, King Fahad Medical City, Riyadh, Saudi Arabia, which officially granted an exemption from full ethical review (reference number 24–245), as the methodology was restricted to gathering anonymous, professional estimations of standard resource utilization without the collection of private personal data. Before Round 1, all experts were informed in writing about the purpose of the elicitation and the use of their responses, volunteered to participate, and all responses were logged anonymously.

## 3. Results

### 3.1. Base-case analysis

The base-case analysis, conducted over a lifetime horizon for a hypothetical cohort of patients with high-risk LACC in Saudi Arabia, found that the addition of pembrolizumab to CCRT was both more effective and costly than CCRT with a placebo. As summarized in Table 4, the total lifetime cost per patient for the pembrolizumab arm was projected to be $466,074.19, compared to $224,030.93 for the placebo arm. This resulted in an incremental cost of $242,043.26. In terms of health outcomes, the pembrolizumab regimen yielded an additional 3.51 Lys and 2.93 QALYs per patient. The combination of these costs and outcomes produced a base-case ICER of $82,626.27 per QALY gained.

**Table 4:** The results of the model’s base-case evaluation.

Group	Costs ($)	ΔCosts ($)	Life-Yr (LYs)	ΔLYs	QALYs	ΔQALYs	ICER ($/QALY)
Pembrolizumab + CCRT	466,074.19	242,043.26	12.08	3.51	10.63	2.93	82,626.27
Placebo + CCRT	224,030.93	–	8.57	–	7.70	–	–

All costs are presented in United States dollars and are discounted along with outcomes.

CCRT = concurrent chemoradiotherapy, ICER = incremental cost-effectiveness ratio, LY = Life-Year, QALY = quality-adjusted life-year.

### 3.2. Sensitivity analyses

To explore the robustness of the base-case results, a series of sensitivity analyses was performed to assess the impact of parameter uncertainty. A one-way sensitivity analysis, illustrated in the tornado diagram (Fig. 3), identified the key drivers of the model outcomes. The analysis confirmed that the ICER was most sensitive to the acquisition cost of pembrolizumab; varying this single parameter caused the ICER to range from approximately $68,000 to over $100,000/QALY. The next most influential parameters were the HSUVs for PD and progression-free disease. In contrast, the model showed negligible sensitivity to other factors, including the costs of terminal care, management of AEs, and probabilities of treatment-related side effects such as anemia and leukopenia.

**Figure 3.**
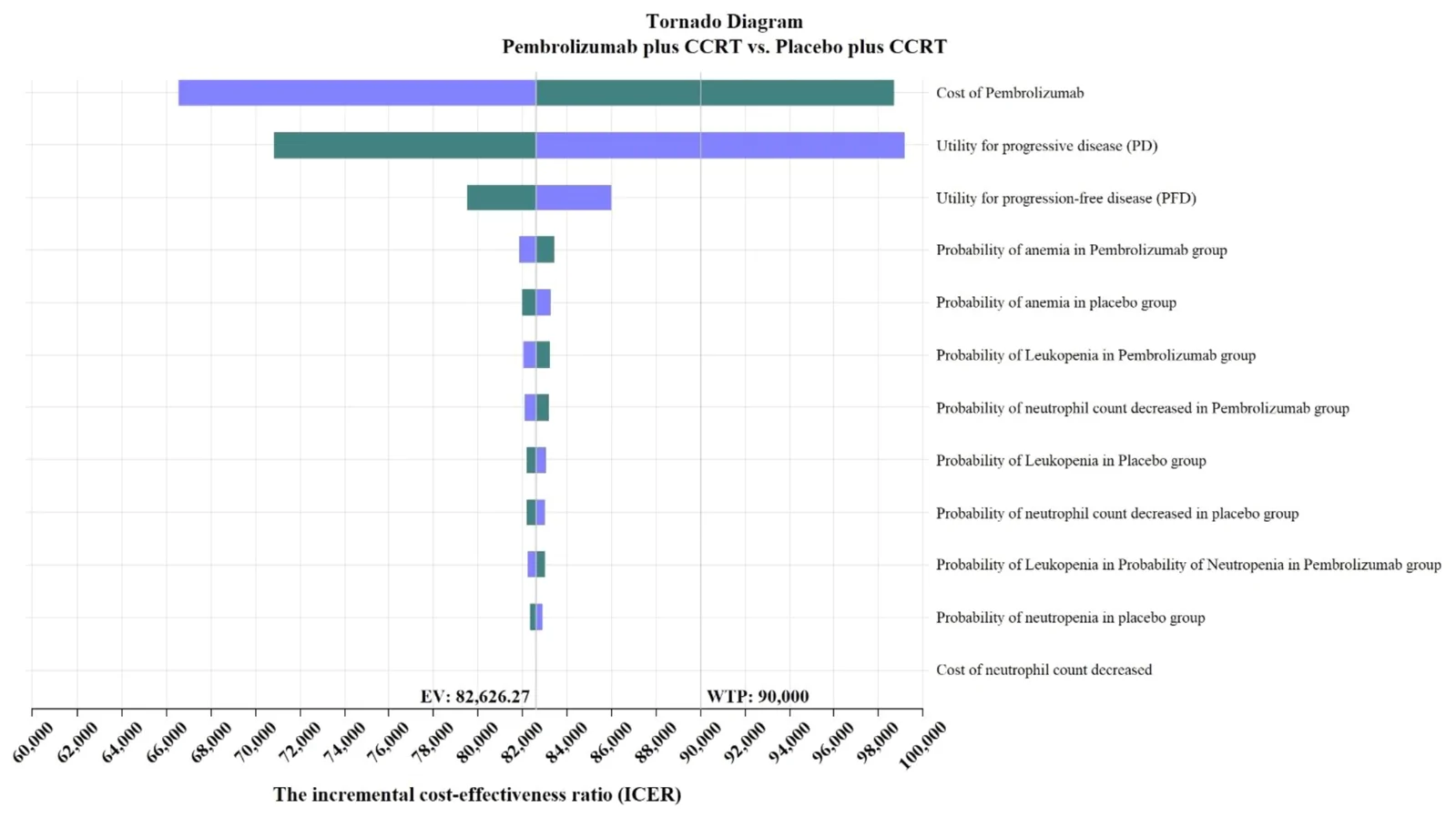
Tornado diagram of the one-way sensitivity analysis.

The results of the scenario analyses are detailed in Supplementary Table S3, Supplemental Digital Content 3. When adopting a societal perspective, the inclusion of productivity savings resulted in a more favorable cost-effectiveness profile, lowering the ICER to $68,422 per QALY. Conversely, the exclusion of SEE-derived costs had a negligible impact, marginally increasing the ICER to $83,102 per QALY, confirming that the model’s conclusions are not driven by expert-elicited parameters. The threshold price analysis indicated that while the current list price is cost-effective at the $90,000/QALY benchmark, substantial discounts would be required to meet lower thresholds. Specifically, to achieve cost-effectiveness at $50,000 and $20,000 per QALY, the acquisition cost of pembrolizumab would need to be reduced by 43.1% and 82.3%, respectively.

CEAC, presented in Figure 4A, further illustrates the cost-effectiveness of the pembrolizumab regimen, which remains cost-effective at various WTP thresholds. At the study’s specified WTP benchmark of $90,000 per QALY, the probability that pembrolizumab plus CCRT would be considered cost-effective was 67%. PSA was conducted to evaluate the impact of simultaneous uncertainty across all parameters. The resulting cost-effectiveness scatter plot (Fig. 4B) showed that 100% of the simulation pairs fell within the northeast quadrant, consistently demonstrating that the pembrolizumab regimen was more effective but also more costly than the comparator.

**Figure 4.**
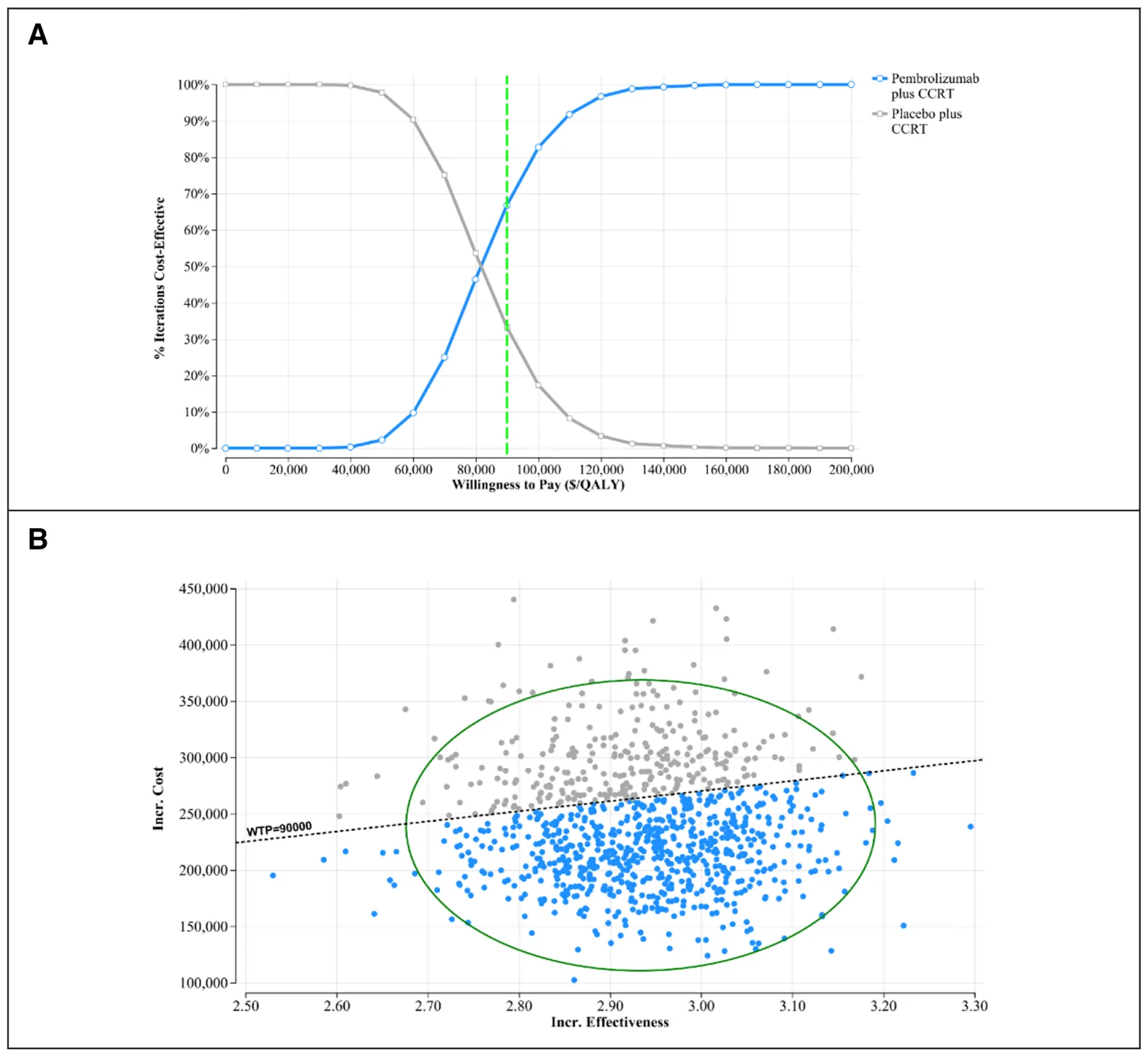
Probabilistic sensitivity analysis results showing (A) the cost-effectiveness acceptability curve and (B) the cost-effectiveness scatter plot.

## 4. Discussion

To our knowledge, this economic evaluation is the first to assess the cost-effectiveness of adding pembrolizumab to standard CCRT for newly diagnosed high-risk LACC from the Saudi Arabian payer perspective. Framed within the national strategic goals of Saudi Vision 2030, which prioritizes both healthcare quality and fiscal sustainability, this analysis addresses a critical policy question. Our base-case analysis demonstrated that the pembrolizumab-containing regimen is a clinically superior intervention. However, the central challenge for policymakers is interpreting this value. The CEAC illustrates that reimbursement decisions are highly dependent on the value policymakers place on the QALY. The probability of cost-effectiveness reached 67% at a threshold of $90,000/QALY; it is approximately 50% at a threshold of $82,000/QALY, indicating that the decision is essentially a coin toss if the threshold is close to the calculated ICER. Conversely, if policymakers’ implicit thresholds were substantially higher, for instance $120,000/QALY, the probability of cost-effectiveness would exceed 95%, providing a much stronger evidence signal to support adoption.

Our base-case ICER of $82,626 per QALY can be externally validated by comparison to similar economic evaluations of the KEYNOTE-A18 regimen. Our findings diverge significantly from those in other countries, highlighting the critical importance of jurisdiction-specific analysis. For instance, a recent analysis from the United States reported a much higher ICER of $183,400 per QALY, concluding the regimen was not cost-effective at a $100,000/QALY threshold without a substantial price reduction.^[14]^ The ICER from our study is considerably lower than that of the United States and the $102,758/QALY reported for Italy, but significantly higher than the $16,217/QALY found in an analysis for China.^[13]^ This dispersion is expected and is primarily driven by local differences in drug acquisition costs: the model’s most sensitive parameter: as well as healthcare system cost structures (e.g., costs for radiotherapy, administration, and AE management). This comparison validates our model’s structure while simultaneously reinforcing our conclusion that cost-effectiveness is not a transferable property; rather, it is a decision contingent on local pricing and policy.

One-way sensitivity analysis clearly demonstrated that the ICER was overwhelmingly sensitive to the price of pembrolizumab. This finding provides a strong evidence-based rationale for policymakers to engage in price negotiations and explore innovative financing models. Given that a key uncertainty is the long-term extrapolation of survival benefits beyond the trial period, we recommend that policymakers at the Ministry of Health, Saudi Food and Drug Authority, and National Unified Procurement Company consider a performance-based risk-sharing agreement for this drug. Under such a scheme, the final reimbursed price for pembrolizumab would be linked to achieving predefined real-world clinical benchmarks, such as PFS rates at 24 or 36 months in a cohort of Saudi patients.^[32]^ This approach would directly address the “efficacy-effectiveness gap” noted in our limitations while mitigating both financial risk and clinical uncertainty for the healthcare system.

Beyond direct price negotiation, these findings strongly support the use of innovative financing models for which the Ministry of Health has already established a formal policy framework. Managed entry agreements are increasingly being viewed in Saudi Arabia as essential strategic tools.^[33]^ However, the successful implementation of such value-based agreements is contingent upon a mature HTA infrastructure, a key component of the Kingdom’s ongoing healthcare transformation. This underscores the parallel need for investment in Saudi-specific real-world data collection through dedicated patient registries, as data availability has been identified as a key challenge in implementing such agreements in the Kingdom.^[34]^ The urgency of these policy actions is magnified by the clinical burden of LACC in Saudi Arabia, where the lack of comprehensive screening programs contributes to a high rate of late-stage diagnosis, making access to clinically superior treatments a national priority.^[35]^ Therefore, this study equips policymakers with the specific data needed to balance profound clinical benefits against budgetary constraints, enabling informed decisions that align with the Kingdom’s long-term strategic health objectives.

This study has several strengths. This is the first cost-effectiveness analysis of this regimen in the Saudi context, directly addressing a critical evidence gap for local policymakers. Our methodology is robust, utilizing a partitioned survival analysis populated with the most current efficacy data from the pivotal KEYNOTE-A18 trial. Furthermore, we enhanced the local relevance of the model by incorporating Saudi Arabia-specific cost data for radiotherapy and conducting a formal SEE to estimate other resource utilization costs, adhering to ISPOR best practices.^[26]^ Comprehensive sensitivity analyses provide a transparent view of decision uncertainty.

This study had several limitations that must also be acknowledged. First, the clinical efficacy data were derived from a global clinical trial; the patient population may not perfectly reflect the real-world characteristics of LACC patients in Saudi Arabia, potentially creating an efficacy-effectiveness gap. Second, owing to the lack of published local data, we used HSUVs from a Korean study.^[27]^ While this study was selected for its methodological alignment (EuroQol 5-Dimension 3-Level instrument) and relevant health state definitions (pre- vs post-progression), cultural differences can influence health state valuations. Our one-way sensitivity analysis (Fig. 3) confirmed that the ICER is highly sensitive to these utility values, identifying this as a key source of uncertainty. This limitation does not invalidate our findings, but it highlights a critical data gap and underscores the urgent, national-level need to develop a Saudi-specific EQ–5D value set to improve the precision of all future local economic evaluations. Third, our analysis utilized publicly listed drug prices, which may not accurately reflect the confidential, discounted prices negotiated by National unified procurement company. Therefore, our base-case ICER should be interpreted as a conservative estimate from the payer’s perspective; the true ICER following confidential price negotiations is likely to be more favorable than our base-case ICER. Fourth, the lack of a formal WTP threshold in Saudi Arabia complicates the definitive interpretation of the results, forcing reliance on generalized international benchmarks. Fifth, radiotherapy costs were sourced from a single large medical center in the capital. Although KAMC functions as a national benchmark, healthcare delivery costs may vary in other hospitals. Consequently, our findings may be most applicable to major comprehensive cancer centers, and caution should be exercised when generalizing the cost results to all healthcare facilities in Saudi Arabia. Finally, the primary base-case analysis was strictly confined to the healthcare payer perspective, thereby deliberately excluding societal costs to align with standard reimbursement assessments. However, incorporating productivity losses averted estimated using the human capital approach yielded a more favorable economic profile. Caregiver time and out-of-pocket costs borne by families were not quantified in any analysis because local data were unavailable; their exclusion may mean the full societal value of the regimen remains understated.

This study highlights several critical areas for future research in Saudi Arabia. There is an urgent need to generate country-specific health state utility data for prevalent diseases, including cervical cancer, to reduce the uncertainty in future economic evaluations of this disease. The formalization of national HTA guidelines, including the establishment of an explicit WTP threshold, would provide a clear and consistent framework for decision-making. Finally, as pembrolizumab is adopted, real-world evidence studies will be invaluable for validating its long-term effectiveness and resource use within the Saudi healthcare system, allowing future refinements of this analysis.

## 5. Conclusion

Based on this economic evaluation, the addition of pembrolizumab to standard CCRT for newly diagnosed high-risk LACC in Saudi Arabia provides substantial clinical benefits but at a high incremental cost, and the pembrolizumab regimen is likely to be considered a cost-effective strategy. While this regimen offers significant therapeutic advancement for a disease with a high unmet need in Saudi Arabia, its value proposition remains highly conditional on its acquisition price. These findings provide crucial evidence to support Saudi policymakers in reimbursement negotiations and inform decisions to ensure sustainable and equitable access to innovative cancer therapies.

## Acknowledgments

The authors extend their appreciation to Prince Sattam bin Abdulaziz University for funding this research work through the project number (PSAU/2025/03/33964).

## Author contributions

**Conceptualization:** Ziyad S. Almalki.

**Data curation:** Ziyad S. Almalki.

**Formal analysis:** Ziyad S. Almalki, Bandar S. Alghamdi, Meshal M. Alqahtani, Nawaf S. Alzahrani, Osama A. Alghamdi, Haitham H. Alanazi.

**Funding acquisition:** Ziyad S. Almalki.

**Investigation:** Ziyad S. Almalki, Bandar S. Alghamdi, Meshal M. Alqahtani, Nawaf S. Alzahrani, Osama A. Alghamdi, Haitham H. Alanazi.

**Methodology:** Ziyad S. Almalki.

**Project administration:** Ziyad S. Almalki.

**Resources:** Ziyad S. Almalki.

**Software:** Ziyad S. Almalki, Ahmad A. Alamer.

**Supervision:** Ziyad S. Almalki.

**Validation:** Ziyad S. Almalki, Ahmad A. Alamer, Shaheed K. Alhumaid.

**Visualization:** Ziyad S. Almalki.

**Writing – original draft:** Ziyad S. Almalki, Shaheed K. Alhumaid.

**Writing – review & editing:** Ahmad A. Alamer, Shaheed K. Alhumaid, Abdulrahman A. Alsuhibani, Bandar S. Alghamdi, Meshal M. Alqahtani, Nawaf S. Alzahrani, Osama A. Alghamdi, Haitham H. Alanazi.






