# Supplementary material for: Cost-effectiveness of pembrolizumab plus chemoradiotherapy versus placebo plus chemoradiotherapy for high-risk locally advanced cervical cancer in Saudi Arabia: A model-based economic evaluation
Source: Medicine (Baltimore). 2026 Oct 2;105(40):e50990. doi: 10.1097/MD.0000000000050990 (PMC13633037; doi:10.1097/MD.0000000000050990)
Supplement: Supplementary file 1 [file medi-105-e50990-s001.docx]

Table S1. Numerical Internal Validation: Model-Predicted vs. Trial-Observed Survival Rates

| **Endpoint** | **Treatment Arm** | **Observed Trial Rate (95% CI)** | **Model-Predicted Rate (Derived)** | **Absolute Difference** | **Relative Deviation** |
| --- | --- | --- | --- | --- | --- |
| **24-Month PFS** | Pembrolizumab + CCRT | 68.0% (62%–73%) | 67.9% | -0.1% | -0.15% |
| **24-Month PFS** | Placebo + CCRT | 57.0% (51%–63%) | 57.3% | +0.3% | +0.53% |
| **36-Month OS** | Pembrolizumab + CCRT | 82.6% (78.4%–86.1%) | 82.5% | -0.1% | -0.12% |
| **36-Month OS** | Placebo + CCRT | 74.8% (70.1%–78.8%) | 74.6% | -0.2% | -0.27% |

Note: PFS = Progression-Free Survival; OS = Overall Survival; CI = Confidence Interval; CCRT = Concurrent Chemoradiotherapy. Observed trial rates are sourced from the KEYNOTE-A18 second interim analysis (for 24-month PFS) and the final analysis presented at ASCO 2025 (for 36-month OS). Model-predicted rates are the deterministic outputs from the calibrated Gompertz (intervention) and Weibull (comparator) parametric survival models used in the base-case analysis.
