# Supplementary material for: Cost-effectiveness of pembrolizumab plus chemoradiotherapy versus placebo plus chemoradiotherapy for high-risk locally advanced cervical cancer in Saudi Arabia: A model-based economic evaluation
Source: Medicine (Baltimore). 2026 Oct 2;105(40):e50990. doi: 10.1097/MD.0000000000050990 (PMC13633037; doi:10.1097/MD.0000000000050990)
Supplement: Supplementary file 2 [file medi-105-e50990-s002.docx]

Table S2. Summary Statistics for Structured Expert Elicitation (SEE) Cost Parameters (in 2025 USD)

| **Parameter Name** | **Mean (Base Case)** | **Min** | **Max** | **Distribution Type** | **Derived SD*** |
| --- | --- | --- | --- | --- | --- |
| **Management of severe events (per event)** |  |  |  |  |  |
| Anemia (Grade 3+) | $1,371.15 | $708.15 | $2,034.15 | Beta-PERT | $221.00 |
| Leukopenia (Grade 3+) | $1,108.02 | $766.02 | $1,450.02 | Beta-PERT | $114.00 |
| Neutropenia (Grade 3+) | $2,076.54 | $1,569.54 | $2,583.54 | Beta-PERT | $169.00 |
| Neutrophil count decreased (Grade 3+) | $2,076.54 | $1,735.54 | $2,617.54 | Beta-PERT | $147.00 |
| **Routine Follow-up (per cycle)** | $685.35 | $320.87 | $1,049.84 | Beta-PERT | $121.49 |
| **Terminal Care (one-off cost)** | $2,256.45 | $734.79 | $2,404.13 | Beta-PERT | $278.22 |

*Note: USD = United States Dollars; SD = Standard Deviation. *Standard Deviation for the Beta-PERT distribution calculated as (Max - Min) / 6.*
