# Supplementary material for: Cost-effectiveness of pembrolizumab plus chemoradiotherapy versus placebo plus chemoradiotherapy for high-risk locally advanced cervical cancer in Saudi Arabia: A model-based economic evaluation
Source: Medicine (Baltimore). 2026 Oct 2;105(40):e50990. doi: 10.1097/MD.0000000000050990 (PMC13633037; doi:10.1097/MD.0000000000050990)
Supplement: Supplementary file 3 [file medi-105-e50990-s003.docx]

Table S3. Summary of Scenario Analyses

| **Panel A: Scenario Analysis** |  | | | |  |
| --- | --- | --- | --- | --- | --- |
| **Scenario / Perspective** | **ΔCosts ($)** | **ΔQALYs** | | **ICER ($/QALY)** | **Change from Base Case** |
| **Base Case (Healthcare Payer)** | **242,043** | **2.93** | | **$82,626** | **Reference** |
| Scenario 1: Societal Perspective* | 200,476 | 2.93 | | $68,422 | -17.2% (More Favorable) |
| Scenario 2: Exclusion of SEE Costs** | 243,488 | 2.93 | | $83,102 | +0.5% (Minimal Impact) |
| **Panel B: Threshold Price Analysis** |  | |  | |  |
| **WTP Threshold Target** | **Description** | | **Required Price Reduction** | | **Resulting Price ($/mg)** |
| $90,000 / QALY | Evaluative Benchmark (WHO-derived) | | None (Current Price) | | $39.20 |
| $50,000 / QALY | Intermediate Benchmark | | 43.1% | | $22.30 |
| $20,000 / QALY | Strict Threshold | | 82.3% | | $6.94 |

* Societal perspective estimates indirect costs using the Human Capital Approach based on 2024 GASTAT data.

** Scenario 2 sets all costs derived from Structured Expert Elicitation (SEE)—including adverse event management and routine follow-up—to zero to test model robustness.
